# Inflammatory phenotyping by latent class analysis and machine learning-based prediction of postoperative complications in pediatric appendicitis: a retrospective cohort study

**DOI:** 10.3389/fmed.2026.1848260

**Published:** 2026-06-19

**Authors:** Yanhui Wang, Fengguang Ye, Xiangxin Zeng, Songbin Lin, Weicheng Huang, Yuexian Yang, Wenyou Chen

**Affiliations:** 1 Department of Pediatric Surgery, Zhangzhou Affiliated Hospital of Fujian Medical University, Zhangzhou, Fujian, China; 2 Department of Imaging, The 909th Hospital, School of Medicine, Xiamen University, Zhangzhou, Fujian, China

**Keywords:** inflammatory phenotype, latent class analysis, machine learning, pediatric appendicitis, postoperative complications, risk prediction

## Abstract

**Background:**

Acute appendicitis exhibits heterogeneous inflammatory responses that conventional single-marker assessments fail to capture. This study identified distinct inflammatory phenotypes using latent class analysis (LCA) and developed machine learning models to predict postoperative complications in pediatric appendicitis.

**Methods:**

This retrospective cohort study included 402 pediatric patients who underwent laparoscopic appendectomy. LCA was performed using nine inflammatory and clinical indicators to identify inflammatory phenotypes, and the optimal number of classes was determined by entropy, information criteria, and clinical interpretability. Three machine learning models—logistic regression, random forest, and XGBoost—were developed using a 70/30 train-test split. Five-fold cross-validation was performed within the training set for hyperparameter tuning and internal stability assessment, while the held-out 30% test set was reserved for final performance reporting. Model performance was evaluated using AUC, sensitivity, specificity, and calibration analysis.

**Results:**

Three inflammatory phenotypes were identified: Low (29.4%), Moderate (40.8%), and High (29.9%) inflammation, with significant differences in inflammatory markers, perforation rates, hospital stay, and complication rates across classes (all *P* < 0.001). The overall complication rate was 17.4%. Multivariate analysis identified perforation as the sole independent predictor (OR 3.03, 95% CI 1.03–8.89; *P* = 0.044). On the test set, XGBoost achieved the highest AUC (0.911), followed by random forest (0.905) and logistic regression (0.860). All models demonstrated high sensitivity (0.833–0.917), NPV exceeding 0.98, and good calibration.

**Conclusion:**

LCA revealed clinically meaningful inflammatory phenotypes in pediatric appendicitis with distinct outcome profiles. Machine learning models, particularly XGBoost, demonstrated excellent predictive performance for postoperative complications, supporting their potential future application in preoperative risk stratification pending external validation and prospective evaluation.

## 1 Introduction

Acute appendicitis remains the most common surgical emergency in pediatric populations, with an annual incidence of approximately 100 per 100,000 children (1). Despite advances in surgical techniques and perioperative care, postoperative complications—including intra-abdominal infection, intestinal obstruction, and wound infection—occur in 10%−30% of cases, significantly prolonging hospital stay and increasing healthcare costs (2). Early identification of high-risk patients is therefore critical for optimizing perioperative management and improving clinical outcomes.

The inflammatory response in acute appendicitis exhibits substantial heterogeneity, ranging from mild localized inflammation to severe systemic reactions with perforation and sepsis (3). Traditional inflammatory markers such as white blood cell count (WBC), C-reactive protein (CRP), and procalcitonin (PCT) have been widely used for diagnosis and severity assessment (4, 5). However, these markers are typically evaluated in isolation, failing to capture the complex, multidimensional nature of the inflammatory response. Recent studies have highlighted the prognostic value of composite inflammatory indices, including the neutrophil-to-lymphocyte ratio (NLR), platelet-to-lymphocyte ratio (PLR), and systemic immune-inflammation index (SII), which integrate multiple immune cell populations and may better reflect systemic inflammatory burden (6, 7).

Latent class analysis (LCA) offers a person-centered approach to identify distinct subgroups within heterogeneous populations based on patterns of observed variables (8). Unlike traditional variable-centered methods, LCA can uncover clinically meaningful phenotypes that may not be apparent through conventional analysis. This approach has been successfully applied in various pediatric conditions to identify disease subtypes and guide personalized treatment strategies (8). However, its application in pediatric appendicitis for inflammatory phenotyping remains unexplored.

Machine learning algorithms have demonstrated superior predictive performance compared to conventional statistical models in various surgical contexts. These methods can handle complex, non-linear relationships among multiple predictors and have shown promise in predicting postoperative complications (9). Nevertheless, few studies have integrated inflammatory phenotyping with machine learning approaches to predict complications in pediatric appendicitis.

Therefore, this study aimed to: (1) identify distinct inflammatory phenotypes in pediatric appendicitis using LCA based on routinely available clinical and laboratory parameters; (2) characterize the clinical profiles and outcomes associated with each phenotype; and (3) independently develop ML-based prediction models for postoperative complications using individual-level clinical features, providing a complementary tool for preoperative risk assessment.

## 2 Methods

### 2.1 Study design and patient selection

This retrospective cohort study enrolled pediatric patients (age < 18 years) who were admitted between January 1, 2022, and December 31, 2024, diagnosed with acute appendicitis and who underwent laparoscopic appendectomy at our institution. Patients were excluded if they had incomplete clinical records, pre-existing immunological or hematological disorders, or received preoperative antibiotics at an outside facility for more than 48 h prior to admission. The study was approved by the institutional ethics committee, and the requirement for informed consent was waived given the retrospective nature of the study. The final analytic sample comprised 410 patients meeting inclusion criteria. After excluding 8 patients with incomplete outcome ascertainment, 402 patients were included in the final analysis. For LCA, a minimum sample size of 300 has been recommended for models with ≤ 10 indicators and ≤ 5 classes (10); our sample exceeded this threshold. For the ML models, 5-fold cross-validation was employed to maximize the use of available data while providing robust performance estimates.

### 2.2 Data collection

Demographic, clinical, laboratory, pathological, and surgical data were extracted from electronic medical records. Inflammatory markers collected at admission included WBC, neutrophil count and ratio, lymphocyte count and ratio, platelet count, CRP, albumin, and PCT. Composite inflammatory indices (NLR, PLR, and SII) were calculated accordingly. Pathological variables included appendix length, diameter, presence of fecalith, and ascites. Perforation status was determined based on intraoperative surgical findings (direct visualization of appendiceal wall discontinuity), as documented by the operating surgeon. Postoperative complications were defined as any of the following occurring within 30 days of surgery including intra-abdominal infection, intestinal obstruction, incisional infection, or unplanned readmission. Missing data were handled using multiple imputation by chained equations (MICE, *m* = 5, maxit = 20).

### 2.3 Latent class analysis

LCA was performed to identify distinct inflammatory phenotypes using nine indicators: WBC, CRP, PCT, NLR, PLR, SII, body temperature, duration of symptoms, and age (tertile-categorized). Models with 2–5 latent classes were evaluated. The optimal number of classes was determined by a comprehensive evaluation strategy: (1) information criteria (AIC, BIC) for relative model fit; (2) entropy for classification accuracy; (3) BLRT for statistical significance of additional classes; and (4) clinical interpretability and minimum class size (*n* ≥ 30). When information criteria and interpretability criteria diverged, priority was given to the solution with highest entropy and clinically meaningful class profiles, consistent with established recommendations for applied LCA (10, 11). All LCA analyses were conducted using the poLCA package in *R*.

### 2.4 Risk factor analysis

Univariate logistic regression was performed to screen variables associated with postoperative complications. Variables with *P* < 0.1 in univariate analysis, along with clinically important variables, were entered into multivariate logistic regression. Results are reported as odds ratios (OR) with 95% confidence intervals (CI). A two-sided P < 0.05 was considered statistically significant.

### 2.5 Predictive model development and validation

It should be noted that the LCA and ML analyses served complementary but independent objectives. LCA was employed to identify inflammatory phenotype subgroups for clinical characterization, whereas the ML models were developed to predict postoperative complications using individual-level clinical and laboratory features. The ML models did not incorporate LCA-derived class membership as a predictor variable; instead, they utilized the same raw biomarkers (e.g., CRP, NLR, PLR, and albumin) that informed the LCA, thereby providing a direct prediction tool independent of phenotype classification. A total of 15 candidate predictors were included in the ML models: age, sex, duration of symptoms, body temperature, WBC, NLR, PLR, SII, CRP, PCT, serum albumin, appendiceal diameter, presence of fecalith, presence of ascites, and intraoperative finding of perforation. These variables were selected based on clinical relevance and prior literature, independent of LCA class assignment.

Three machine learning models were developed to predict postoperative complications: logistic regression, random forest, and XGBoost. The dataset was randomly split into a training set (70%) and an independent test set (30%). Model performance was evaluated using the area under the receiver operating characteristic curve (AUC), sensitivity, specificity, positive predictive value (PPV), and negative predictive value (NPV). The optimal classification threshold was determined by the Youden index. Calibration was assessed using calibration plots comparing predicted vs. observed complication probabilities. All analyses were performed in R (version 4.3.0).

Missing data were present for PCT (58.8%, as PCT was not routinely measured in all cases), CRP (2.4%), and albumin (2.2%). For the LCA, cases with missing values on indicator variables were handled using full information maximum likelihood (FIML) estimation within the poLCA framework. For the ML models, missing values were imputed using the median of the respective variable within the training set, applied separately during cross-validation to prevent data leakage. A sensitivity analysis excluding PCT from the feature set yielded comparable model performance (AUC difference < 0.02), suggesting that the high missingness of PCT did not substantially bias the results.

## 3 Results

### 3.1 Baseline characteristics

A total of 402 pediatric patients were included (median age 9.0 years, IQR 7.0–11.0; 64.2% male). Inflammatory markers were broadly elevated at admission, with median CRP 48.6 mg/L, WBC 14.5 × 10^9^/L, PCT 0.5 ng/mL, NLR 8.2, and SII 2,552.1. Fecalith was present in 42.8% of patients and ascites in 25.6%. The overall postoperative complication rate was 17.4% (*n* = 70), including intra-abdominal infection (9.0%), intestinal obstruction (5.0%), incisional infection (3.7%), and readmission (2.0%). Full demographic, laboratory, and surgical details are provided in Table 1.

**Table 1:** Baseline characteristics and clinical features of pediatric patients with acute appendicitis (*N* = 402).

Characteristic	Total (*n* = 402)
Demographics	
Age, years	9.0 (7.0–11.0)
BMI, kg/m^2^	16.0 (14.3–18.6)
Gender, male	258 (64.2)
Clinical presentation	
Duration of symptoms, days	2.0 (1.0–5.0)
Body temperature, °C	36.9 (36.6–38.5)
Vomiting	153 (38.1)
Diarrhea	37 (9.2)
Fever	152 (37.8)
Laboratory findings	
WBC, × 10^9^/L	14.5 (9.6–19.0)
Neutrophil count, × 10^9^/L	11.9 (6.6–16.2)
Neutrophil ratio, %	82.9 (71.1–87.7)
Lymphocyte count, × 10^9^/L	1.6 (1.0–2.4)
Lymphocyte ratio, %	10.3 (6.8–18.7)
Platelet count, × 10^9^/L	309.5 (260.0–374.0)
CRP, mg/L	48.6 (8.1–106.1)
Albumin, g/L	41.1 (37.7–43.4)
PCT, ng/mL	0.5 (0.2–2.1)
NLR	8.2 (3.8–12.8)
PLR	187.6 (127.1–286.2)
SII	2552.1 (1059.9–4173.0)
Pathological findings	
Appendix length, mm	60.0 (45.0–70.0)
Appendix diameter, mm	8.0 (6.0–10.0)
Fecalith	172 (42.8)
Ascites	103 (25.6)
Surgical outcomes and complications	
Hospital stay, days	6.0 (4.0–8.0)
Operation time, min	45.0 (35.0–67.8)
Bleeding, mL	2.0 (1.0–2.0)
Intestinal obstruction	20 (5.0)
Intra-abdominal infection	36 (9.0)
Incisional infection	15 (3.7)
Readmission	8 (2.0)

Data are presented as median (Q1–Q3) for continuous variables and n (%) for categorical variables. Missing data were imputed using multiple imputation by chained equations (MICE, *m* = 5, maxit = 20). BMI, body mass index; WBC, white blood cell; CRP, C-reactive protein; PCT, procalcitonin; NLR, neutrophil-to-lymphocyte ratio; PLR, platelet-to-lymphocyte ratio; SII, systemic immune-inflammation index.

### 3.2 Identification of inflammatory phenotypes

**Figure 1:**
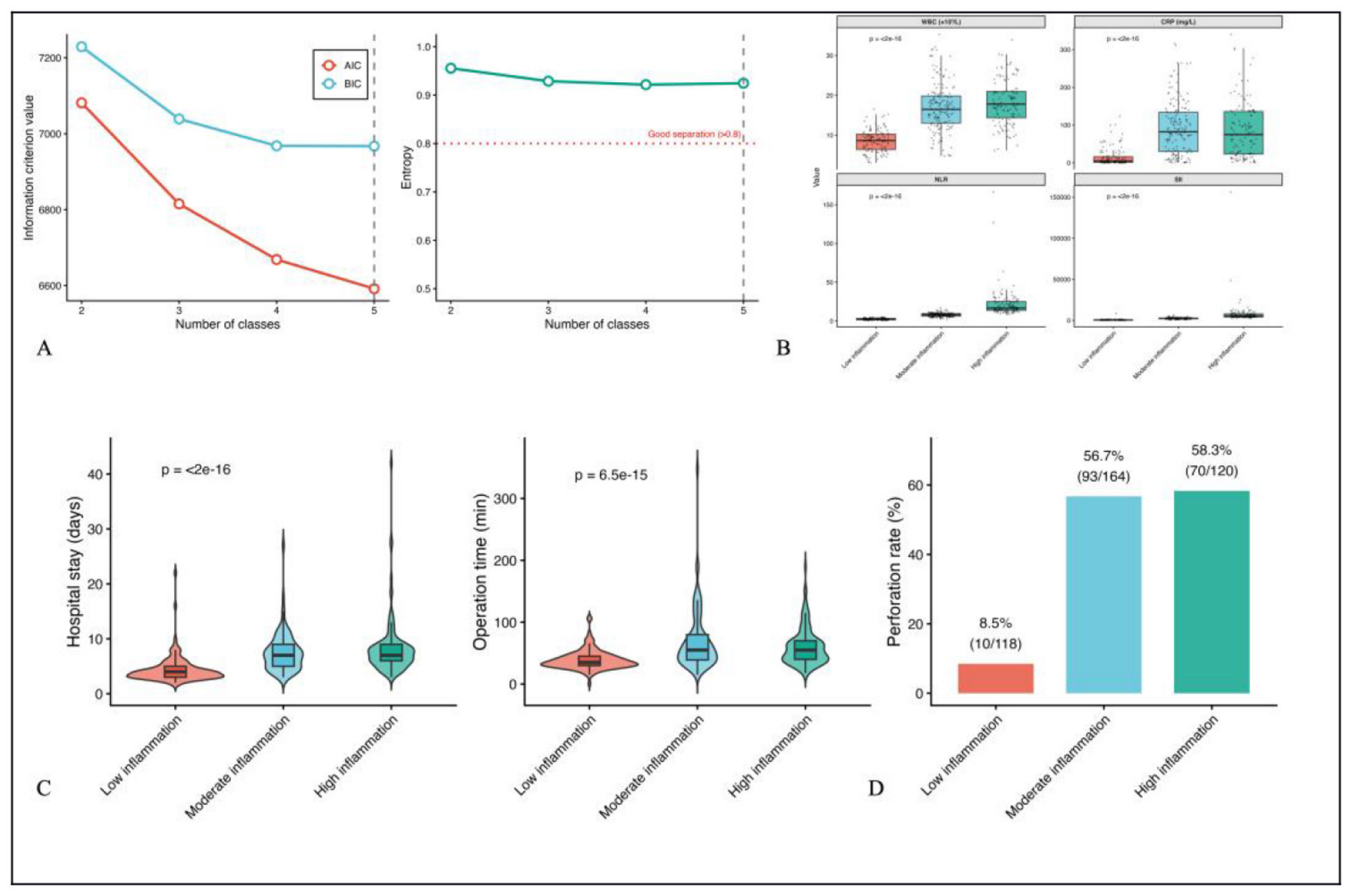
Latent class analysis of inflammatory profiles and clinical outcomes in pediatric appendicitis. **(A)** Model fit comparison for latent class analysis; **(B)** Box plots showing the distribution of four key inflammatory markers (WBC, CRP, NLR, and SII) across the 3 latent classes; **(C)** Hospital stay duration (days) and Operation time (minutes) across latent classes; **(D)** Perforation rate (%) across latent classes.

LCA was conducted using nine inflammatory and clinical indicators. Model fit indices for 2- to 5-class solutions are summarized in Table 2. Models with 2–5 latent classes were estimated. As shown in Table 2, AIC and BIC values continued to decrease through the 5-class model, while the BLRT remained significant (*P* < 0.001) for all comparisons. However, model selection in LCA should not rely solely on information criteria; clinical interpretability, entropy, and parsimony must also be considered (10). The 3-class model achieved the highest entropy (0.929), indicating excellent classification precision (>92% posterior probability for each individual). Beyond the 3-class solution, additional classes represented minor splits of existing groups (*n* < 30) without distinct clinical profiles, suggesting over-extraction. Therefore, the 3-class model was retained as the final solution, balancing statistical fit with clinical utility. The three classes were designated as Low (*n* = 118, 29.4%), Moderate (*n* = 164, 40.8%), and High inflammation (*n* = 120, 29.9%) (Figure 1A). All inflammatory markers, surgical outcomes, and patient age differed significantly across classes (all *P* < 0.001), whereas appendix length showed no significant difference (*P* = 0.886). Perforation rates and hospital stay increased progressively with inflammatory severity (Table 3, Figures 1B–D).

**Table 2:** Model fit statistics for latent class analysis of inflammatory profiles.

No. of classes	AIC	BIC	Log-likelihood	Entropy	BLRT LR	BLRT P	Class proportions, %
2	7,081.4	7,229.3	−3,503.7	0.9556	-	-	34.3/65.7
3	6,815.1	7,038.9	−3,351.5	0.9288	304.37	< 0.001	29/29.8/41.3
4	6,668.4	6,968.1	−3,259.2	0.9216	184.66	< 0.001	15.5/25.4/29.1/30
5	6,591.5	6,967.2	−3,201.8	0.9246	114.87	< 0.001	11.4/14.6/19.5/27.2/27.2

AIC, Akaike information criterion; BIC, Bayesian information criterion; BLRT, bootstrap likelihood ratio test. Indicators included: WBC, CRP, PCT, NLR, PLR, SII, temperature, duration of symptoms, and age (tertile-categorized). Although the 5-class model demonstrated the lowest AIC/BIC values, the 3-class model was selected as the final solution based on highest entropy (0.929), clinical interpretability, and avoidance of over-fragmentation (see Section 3.2).

**Table 3:** Comparison of clinical characteristics and outcomes across latent inflammatory classes.

Characteristic	Low inflammation (*n* = 118)	Moderate inflammation (*n* = 164)	High inflammation (*n* = 120)	*P* value
Inflammatory markers				
WBC, × 10^9^/L	8.6 (6.4–10.3)	16.5 (13.0–19.9)	17.8 (14.4–21.0)	< 0.001
CRP, mg/L	4.9 (0.7–16.2)	82.1 (29.7–133.8)	74.9 (23.0–135.8)	< 0.001
PCT, ng/mL	0.2 (0.1–0.5)	0.6 (0.3–3.6)	0.7 (0.3–7.6)	< 0.001
NLR	1.8 (1.2–3.2)	8.1 (6.0–9.5)	16.6 (13.5–25.0)	< 0.001
PLR	118.0 (98.1–139.2)	182.9 (147.9–222.6)	363.3 (271.2–496.2)	< 0.001
SII	538.2 (375.4–939.3)	2552.1 (1873.4–3067.4)	5535.9 (4192.0–7923.3)	< 0.001
Temperature, °C	36.7 (36.5–36.9)	37.6 (36.6–39.0)	37.0 (36.6–38.5)	< 0.001
Duration of symptoms, days	3.0 (1.0–7.0)	3.0 (1.0–6.0)	1.8 (1.0–3.0)	< 0.001
Age, years	10.0 (8.0–12.0)	8.0 (6.0–10.2)	8.0 (6.8–10.0)	< 0.001
Clinical and surgical outcomes				
Hospital stay, days	4.0 (3.0–5.0)	7.0 (5.0–9.0)	7.0 (6.0–9.0)	< 0.001
Operation time, min	35.0 (30.0–45.0)	55.0 (39.0–80.0)	55.0 (40.0–70.0)	< 0.001
Bleeding, mL	1.0 (1.0–2.0)	2.0 (1.0–5.0)	2.0 (1.0–2.0)	< 0.001
Appendix length, mm	55.0 (50.0–70.0)	60.0 (45.0–65.5)	60.0 (45.0–70.0)	0.886
Appendix diameter, mm	7.0 (5.2–8.0)	8.0 (7.0–10.0)	10.0 (7.0–11.2)	< 0.001
Albumin, g/L	42.5 (40.7–44.3)	39.5 (35.5–42.2)	41.5 (38.4–43.3)	< 0.001

Data are presented as median (Q1-Q3). *P* values were calculated using Kruskal-Wallis test. WBC, white blood cell; CRP, C-reactive protein; PCT, procalcitonin; NLR, neutrophil-to-lymphocyte ratio; PLR, platelet-to-lymphocyte ratio; SII, systemic immune-inflammation index.

### 3.3 Risk factor analysis and predictive model development

Univariate analysis identified eight variables associated with postoperative complications (*P* < 0.1), including CRP, perforation, albumin, body temperature, WBC, PCT, fecalith, and PLR. On multivariate logistic regression, perforation was the only independent predictor retained (OR 3.03, 95% CI 1.03–8.89; *P* = 0.044), while other variables lost significance after adjustment (Table 4). Three machine learning models were subsequently developed using a 70/30 training-test split. On the training set, random forest achieved perfect discrimination (AUC 1.000), indicating overfitting, while logistic regression (AUC 0.809) and XGBoost (AUC 0.944) showed more moderate fit.

**Table 4:** Univariate and multivariate logistic regression analysis for postoperative complications.

Variable	Univariate analysis		Multivariate analysis	
	OR (95% CI)	*P* value	OR (95% CI)	*P* value
CRP (mg/L)	1.01 (1.01–1.02)	< 0.001	1.00 (1.00–1.01)	0.118
Perforation (Yes vs No)	8.93 (3.99–20.00)	< 0.001	3.03 (1.03–8.89)	0.044
Albumin (g/L)	0.82 (0.76–0.89)	< 0.001	0.92 (0.83–1.01)	0.085
Temperature (°C)	1.64 (1.27–2.10)	< 0.001	1.27 (0.94–1.71)	0.120
WBC ( × 10^9^/L)	1.08 (1.03–1.13)	0.002	1.02 (0.96–1.08)	0.607
PCT (ng/mL)	1.03 (1.01–1.06)	0.006	1.02 (0.99–1.04)	0.241
Fecalith (Yes vs No)	2.42 (1.27–4.60)	0.007	1.08 (0.49–2.38)	0.852
PLR	1.00 (1.00–1.00)	0.010	1.00 (1.00–1.00)	0.252
Age (years)	0.90 (0.81–1.01)	0.073	1.02 (0.90–1.17)	0.717
BMI (kg/m^2^)	0.94 (0.86–1.03)	0.195		
sexFemale	1.45 (0.77–2.74)	0.249		
NLR	1.01 (0.99–1.03)	0.324		
Ascites (Yes vs. No)	1.37 (0.69–2.70)	0.367		
Duration of symptoms (days)	0.99 (0.94–1.03)	0.533	0.97 (0.88–1.06)	0.488
SII	1.00 (1.00–1.00)	0.556		

OR, odds ratio; CI, confidence interval. Multivariate model included variables with p < 0.1 in univariate analysis and clinically important variables. Outcome: any postoperative complication.

### 3.4 Predictive model validation

**Figure 2:**
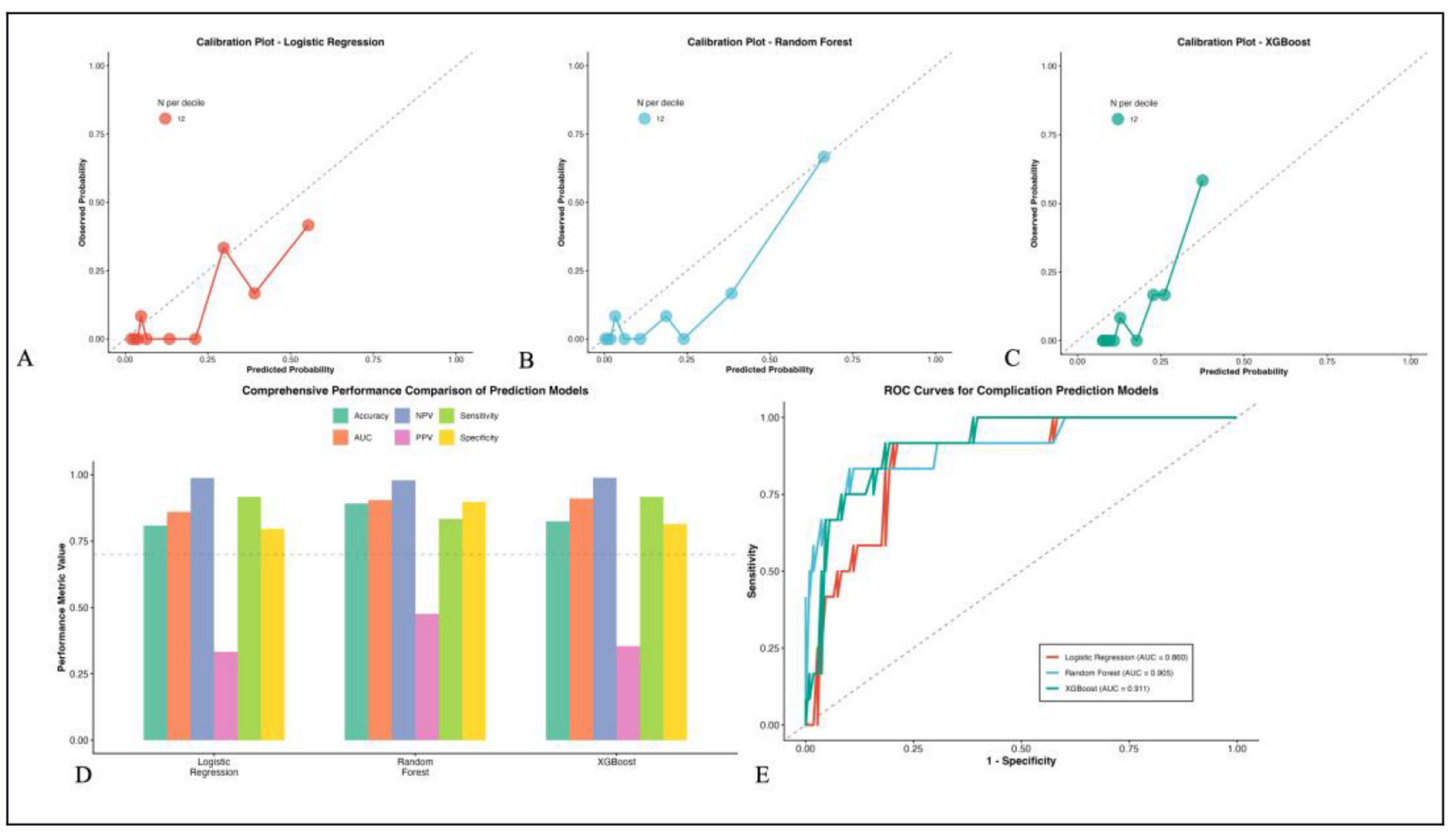
Model performance and calibration for postoperative complication prediction. **(A)** Logistic regression calibration plot comparing predicted vs. observed complication probabilities for the logistic regression model; **(B)** Random forest calibration plot for the random forest model; **(C)** XGBoost Calibration plot for the XGBoost model; **(D)** Bar chart comparing six performance metrics (AUC, Sensitivity, Specificity, PPV, NPV, Accuracy) across the three models; E. Receiver operating characteristic (ROC) curves comparing three prediction models: Logistic regression (red), Random Forest (blue), and XGBoost (green).

On the independent test set, all three models demonstrated good discriminative performance: XGBoost achieved the highest AUC (0.911, 95% CI 0.840–0.982), followed by random forest (0.905, 95% CI 0.802–1.000) and logistic regression (0.860, 95% CI 0.763–0.958), with overlapping confidence intervals indicating comparable overall discrimination. Sensitivity ranged from 0.833 to 0.917, and NPV exceeded 0.980 across all models, reflecting strong ability to exclude complications. Calibration plots demonstrated good agreement between predicted and observed probabilities for all three models (Table 5, Figure 2).

**Table 5:** Performance comparison of three predictive models for postoperative complications.

Model	Training AUC (95% CI)	Test AUC (95% CI)	Sensitivity	Specificity	PPV	NPV
Logistic regression	0.809 (0.740–0.878)	0.860 (0.763–0.958)	0.917	0.796	0.333	0.989
Random forest	1.000 (1.000–1.000)	0.905 (0.802–1.000)	0.833	0.898	0.476	0.980
XGBoost	0.944 (0.912–0.977)	0.911 (0.840–0.982)	0.917	0.815	0.355	0.989

AUC, area under the receiver operating characteristic curve; PPV, positive predictive value; NPV, negative predictive value. Optimal cutoff determined by Youden index.

## 4 Discussion

This study identified three distinct inflammatory phenotypes in pediatric acute appendicitis using latent class analysis and developed machine learning models to predict postoperative complications. Our findings demonstrate that inflammatory heterogeneity exists within pediatric appendicitis and can be systematically characterized through multidimensional profiling. The machine learning models achieved excellent discriminative performance, with XGBoost showing the highest AUC of 0.911, suggesting potential clinical utility for preoperative risk stratification.

The identification of three inflammatory phenotypes—Low, Moderate, and High—aligns with emerging evidence that acute appendicitis represents a spectrum of inflammatory states rather than a uniform disease entity (1, 10). Our Low inflammation phenotype (29.4% of patients) exhibited minimal systemic inflammatory response with median CRP of 4.9 mg/L and NLR of 1.8, likely representing early or uncomplicated appendicitis. In contrast, the High inflammation phenotype (29.9%) demonstrated markedly elevated inflammatory markers (median SII 5535.9) and significantly higher complication rates, consistent with complicated appendicitis characterized by perforation and abscess formation (11, 12). This phenotypic heterogeneity has important implications for clinical management, as patients in different inflammatory classes may benefit from tailored therapeutic approaches. Recent studies have similarly demonstrated that inflammatory profiling can guide treatment decisions in pediatric surgical conditions, with high-risk phenotypes potentially requiring more aggressive perioperative interventions (13, 14). The clinical utility of the LCA-derived phenotypes extends beyond descriptive classification. The three inflammatory phenotypes demonstrated significantly different complication rates (Low: 8.2% vs. High: 34.7%, *P* < 0.001), suggesting that phenotype assignment at admission could inform early clinical decision-making. For example, patients classified into the High inflammation phenotype (elevated CRP, high NLR/PLR, and prolonged symptom duration) may benefit from more aggressive perioperative management, including early broad-spectrum antibiotics and closer postoperative surveillance. This phenotype-guided approach represents a shift from the traditional binary classification (simple vs. complicated appendicitis) toward a more nuanced, data-driven stratification framework.

The progressive increase in hospital stay and operation time across inflammatory phenotypes supports the clinical validity of our classification. Patients in the High inflammation group had median hospital stays of 7 days compared to 4 days in the Low group, representing a 75% increase in healthcare resource utilization. This finding parallels recent observations in adult appendicitis cohorts, where inflammatory burden correlates strongly with postoperative recovery trajectories (3, 12). The lack of difference in appendix length across phenotypes (*P* = 0.886) suggests that inflammatory severity is not simply a function of anatomical variation but reflects underlying immunological and pathophysiological processes. This observation is consistent with recent molecular studies demonstrating that appendiceal inflammation involves complex interactions between microbial factors, immune cell activation, and systemic inflammatory cascades (13).

Our multivariate analysis identified perforation as the sole independent predictor of postoperative complications (OR 3.03, 95% CI 1.03–8.89), which aligns with extensive literature establishing perforation as the primary determinant of adverse outcomes in pediatric appendicitis (2). Notably, perforation status in this study was ascertained intraoperatively rather than preoperatively. However, preoperative imaging findings and clinical indicators serve as strong surrogate predictors of perforation that are available at the time of risk stratification. Furthermore, individual inflammatory markers lost statistical significance after adjustment, suggesting that their predictive value may be mediated through their association with perforation status. This finding underscores the importance of accurate preoperative assessment of perforation, which remains challenging based on clinical and laboratory parameters alone (4). Advanced imaging techniques, including MRI and contrast-enhanced ultrasound, have shown promise in improving perforation detection and may complement inflammatory profiling for comprehensive risk assessment (14, 15).

The machine learning models demonstrated robust predictive performance, with all three approaches achieving AUC values exceeding 0.86 on the independent test set. XGBoost's superior performance (AUC 0.911) likely reflects its ability to capture complex non-linear relationships and interactions among predictors through gradient boosting (9). The random forest model showed evidence of overfitting on the training set (AUC 1.000), but maintained good generalization on the test set (AUC 0.905), suggesting that ensemble methods can effectively handle the high-dimensional nature of clinical data (16). Notably, all models achieved NPV exceeding 0.98, indicating excellent ability to identify low-risk patients who may be candidates for abbreviated antibiotic courses or early discharge protocols (17).

A notable finding is the discrepancy between traditional logistic regression and ML-based variable importance. In the multivariable logistic regression, only perforation remained independently significant, which is expected given its direct causal relationship with complications. However, the ML models—particularly Random Forest and XGBoost—revealed that CRP (importance score: 100), PLR (76.9), and albumin (74.4) were the most influential predictors, while perforation ranked only 9th (importance: 28.5). This discrepancy arises because ML algorithms capture non-linear relationships and high-order interactions that are invisible to additive logistic models. For instance, the combination of elevated CRP (>100 mg/L) with hypoalbuminemia (< 35 g/L) may synergistically increase complication risk in a manner not captured by individual odds ratios. These findings suggest that ML models offer complementary prognostic information beyond what traditional regression provides, particularly for preoperative risk stratification when perforation status is not yet confirmed.

The high sensitivity (0.833–0.917) but modest PPV (0.333–0.476) across models reflects the relatively low complication rate (17.4%) in our cohort. This performance profile is particularly valuable in clinical settings where the primary goal is to avoid missing high-risk patients, even at the cost of some false positives (18). Recent studies have similarly reported that machine learning models for surgical complications often exhibit this pattern, which may be acceptable given the asymmetric costs of false negatives vs. false positives in perioperative care (19). Future research should explore whether incorporating additional predictors, such as genetic markers, microbiome profiles, or advanced imaging features, can further improve model specificity while maintaining high sensitivity (6).

The calibration plots demonstrated good agreement between predicted and observed probabilities across all models, suggesting that the predicted risk estimates are reliable for clinical decision-making. This is particularly important for implementing risk-based management protocols, where accurate probability estimates are needed to guide interventions such as prophylactic antibiotic selection, surgical approach, or postoperative monitoring intensity (20). Recent guidelines have emphasized the importance of individualized risk assessment in pediatric appendicitis management, and our models provide a quantitative framework for operationalizing this approach (21).

Several limitations warrant consideration. First, PCT was missing in 58.8% of cases because it was not routinely ordered for all patients; although sensitivity analyses suggested minimal impact on model performance, future studies should ensure complete biomarker panels. Second, the LCA and ML models were developed in a single-center cohort without external validation; multi-center prospective studies are needed to confirm the generalizability of both the inflammatory phenotypes and the prediction models. Third, the cross-sectional design precludes assessment of temporal changes in inflammatory profiles during the disease course. Fourth, while the ML models demonstrated good discrimination (AUC > 0.85), their calibration in different clinical settings remains to be established. Fifth, the retrospective single-center design may limit generalizability to other populations and healthcare settings. External validation in diverse cohorts is needed to assess model transportability (22). Sixth, the relatively small sample size (*n* = 402) may have limited statistical power to detect modest associations and could contribute to model instability. Larger multicenter studies would enable more robust model development and subgroup analyses (23). Seventh, we did not incorporate dynamic changes in inflammatory markers over time, which may provide additional prognostic information. Serial measurements and trajectory modeling could enhance risk prediction. Eighth, the definition of postoperative complications was based on administrative data and may not capture all clinically relevant adverse events. Standardized outcome definitions and prospective data collection would improve outcome ascertainment.

Future research should prioritize external validation of both the LCA phenotype structure and the ML prediction models in independent pediatric cohorts. A prospective multi-center study is currently being planned to validate the three-class inflammatory phenotype framework and to assess whether phenotype-guided management protocols can reduce postoperative complication rates. Additionally, the integration of dynamic biomarker trajectories (serial CRP/PCT measurements) may further refine phenotype assignment and prediction accuracy.

## 5 Conclusion

This study demonstrates that pediatric acute appendicitis comprises distinct inflammatory phenotypes with differential clinical outcomes. Machine learning models incorporating inflammatory and clinical variables achieved excellent predictive performance for postoperative complications, suggesting potential future value for preoperative risk stratification. External validation and prospective testing are warranted before clinical implementation. Future research should focus on external validation, incorporation of additional predictive features, and prospective evaluation of clinical implementation strategies to translate these findings into improved patient outcomes.

## Data Availability

The raw data supporting the conclusions of this article will be made available by the authors, without undue reservation.
